# The impact of surgical outcome after pancreaticoduodenectomy in elderly patients

**DOI:** 10.1186/1477-7819-9-102

**Published:** 2011-09-11

**Authors:** Yasuhiro Ito, Takeshi Kenmochi, Tomoyuki Irino, Tomohisa Egawa, Shinobu Hayashi, Atsushi Nagashima, Yuko Kitagawa

**Affiliations:** 1 Department of Surgery, Saiseikai Yokohamashi Tobu Hospital, 3-6-1 Shimosueyoshi, Tsurumi-ku, Yokohama-shi, Kanagawa, 230-0012 Japan; 2 Department of Surgery, Keio University School of Medicine, 35 Shinanomachi, Shinjuku-ku, Tokyo, 160-8582 Japan

**Keywords:** Pancreaticoduodenectomy, Elderly patients, Outcome

## Abstract

**Background:**

The elderly population has increased in many countries. Indications for cancer treatment in elderly patients have expanded, because surgical techniques and medical management have improved remarkably. Pancreaticoduodenectomy (PD) requires high-quality techniques and perioperative management methods. If it is possible for elderly patients to withstand an aggressive surgery, age should not be considered a contraindication for PD. Appropriate preoperative evaluation of elderly patients will lead to their safer management. The purpose of the present study was to evaluate the safety of PD in patients older than 75 years and to show the influence of advanced age on the morbidity and mortality associated with this operation.

**Patients and methods:**

Subjects were 98 patients who underwent PD during the time period from April 2005 to April 2011. During this study, 31 patients were 75 years of age or older (group A), and the other 67 patients were less than 75 years old (group B). Preoperative demographic and clinical data, surgical procedure, pathologic diagnosis, postoperative course and complication details were collected prospectively and they were analyzed in two group.

**Results:**

There was no statistical difference between patient groups in terms of gender, comorbidity, preoperative drainage, diagnosis, or laboratory data. Preoperative albumin values were lower in group A (P = 0.04). The mean surgical time in group A was 408.1 ± 73.47 min. Blood loss and blood transfusion were not significantly different between both groups. There was no statistical differences in mortality rate (P = 0.14), morbidity rate (P = 0.43), and mean length of hospital stay (P = 0.22) between both groups.

Long-term survival was also no statistically significant difference between the two groups using the log-rank test (P = 0.10).

**Conclusion:**

It cannot be ignored that the elderly population is getting larger. We must investigate the management of elderly patients after PD and prepare further for more experiences of PD. If appropriate surgical management is provided to elderly patients, we suggest that PD will lead to no adverse effects after surgery, and PD can be performed safely in elderly patients. We conclude that age should not be a contraindication to PD.

## Background

The elderly population has increased in many countries. Indications for cancer treatment in elderly patients have expanded, because surgical techniques and medical management have improved remarkably. However, given that the morbidity and mortality associated with surgical procedures are poorly defined in this population, the decision to perform an operation in an elderly patient can be difficult [1]. Therefore, surgeons must give this decision careful consideration. Pancreaticoduodenectomy (PD), which may cause considerable complications, including pancreatic fistula, intra-abdominal bleeding, intra-abdominal abscess, sepsis and organ failure, requires high-quality techniques and perioperative management methods. Though PD is performed in many hospitals, its associated morbidity and mortality rates are high. In particular, pancreatic fistula still occurs in 5% to 40% of patients, despite refinements in surgical techniques and perioperative management methods [2-5]. In spite of its high rate of morbidity, this aggressive surgery is considered curative. It has been suggested that patients who undergo PD appear to benefit from referral to a high-volume center [6-8]. If it is possible for elderly patients to withstand an aggressive surgery, age should not be considered a contraindication for PD [9]. Appropriate preoperative evaluation of elderly patients will lead to their safer management. In fact, it has been reported that PD can be performed safely in elderly patients [10-12].

The purpose of the present study was to evaluate the safety of PD in patients older than 75 years and to show the influence of advanced age on the morbidity and mortality associated with this operation.

## Methods

### Protocol

Subjects were 98 consecutive patients who underwent PD during the time period from April 2005 to April 2011. The final diagnoses of these patients were Pancreatic carcinoma (n = 39), Cholangiocarcinoma (n = 37), Ampullary carcinoma (n = 8), Intraductal papillary mucinous neoplasm (n = 6), Duodenal carcinoma (n = 3), Gastric carcinoma (n = 1), Duodenal invasion by colon carcinoma (n = 1) and Others (n = 3). The operative technique chosen for pancreatic anastomosis involved drainage of the pancreatic stump into an isolated loop of the jejunum with an end-to-side, duct-to-mucosa anastomosis, including a pancreatic stent in all cases. Two techniques were applied in a strictly alternating way. As a result, the patients were allocated to two groups: (a) an internal stent group that underwent PD involving a pancreatic stump anastomosis to an isolated loop of the jejunum with an end-to-side, duct-to-mucosa anastomosis with an internal stent and (b) an external stent group that underwent PD with an external stent.

### Surgical technique

Conventional or pylorus-preserving PD (PPPD) was performed at the discretion of the individual surgeon. Lymph nodes around the head of the pancreas, the common hepatic artery, and the hepatoduodenal ligament were dissected. Wedge or segmental resection of the portal vein or superior mesenteric vein was performed when the pancreatic head mass was inseparable from the vein. After resection, anastomoses were constructed to a single jejunal loop, which was repositioned up into the supra-mesocolic compartment in a retrocolic manner.

Pancreaticojejunal anastomosis was performed in an end-to-side fashion. The patients were allocated to the internal stent group and the external stent group. After pancreaticojejunal anastomosis, an end-to-side, single layer, interrupted hepaticojejunostomy without stenting was performed. The operation was completed with an end-to-side duodenojejunostomy with mechanical dilation of the pylorus 40 cm downstream from the pancreaticojejunostomy.

### Perioperative management

Twenty-six patients underwent PD, and seventeen patients underwent substomach-preserving PD (SSPPD) with Child reconstruction. Fifty-five patients underwent PPPD with Traverso reconstruction. Pancreatic anastomosis after PD, SSPPD and PPPD was performed by duct-to-mucosal, end-to-side pancreaticojejunostomy in all enrolled patients.

Perioperative management was standardized. All patients received broad-spectrum antibiotics for two days and an H2 blocker (famotidine) during the entire postoperative hospital course. No prophylactic somatostatin or Octreotide was used. The nasogastric tube was removed on the first postoperative day when discharge was less than 500 ml. The volume of fluid drained from the peripancreatic drains and from the pancreatic duct was measured daily. Patients were kept nil per os for the first five postoperative days, after which the diet was gradually resumed if there was no evidence of delayed gastric emptying, pancreatic leakage or other intra-abdominal complications. Total parenteral nutrition was used only in patients who could not tolerate a diet after postoperative day five. The peripancreatic drains were removed if there was no evidence of leakage. If there was evidence of leakage or suspicion of infective complications (fever, leukocytosis or purulent drain fluid), the peripancreatic drains were left in-situ, and a contrast computed tomography (CT) scan was performed to look for any intra-abdominal collection.

Patients were discharged with the pancreatic duct catheter in-situ, and this was removed at our outpatient clinic after the fourth postoperative week.

### Data collection

Preoperative demographic and clinical data, surgical procedure, pathologic diagnosis, postoperative course and complication details were collected prospectively.

### Statistical analysis

Continuous data are expressed as mean ± standard deviation (SD). The χ2 test was used to compare qualitative parameters, and Student's t-test was used for quantitative parameters. Patient overall survival was evaluated using the Kaplan-Meier method and compared with the log-rank test. P < .05 was considered significant.

## Results

### Patient characteristics

PDs were performed in 98 patients at our institution between April 2005 and April 2011. During this study, 31 patients (31.6%) were 75 years of age or older (group A), and the other 67 patients (68.4%) were less than 75 years old (group B). There was no statistical difference between patient groups in terms of gender, comorbidity, preoperative drainage, diagnosis, or laboratory data (hemoglobin, total bilirubin, amylase, hemoglobin A1c). Preoperative albumin values were lower in group A than in group B (P = .04). Patient characteristics and preoperative laboratory datas according to age group are shown in Table 1. Of 90 total patients over 75 years that we saw, we decided not to perform surgery for 59 (65.6%). PD was decided for all of the elderly patients for whom surgery could be performed. In our institution, the indication for PD is resectable cases, as well as younger patients. PD was performed in the absence of peritoneal or distant metastases and when a tumor was not locally advanced. Cases with limited invasion of a portal or superior mesenteric vein were considered to be resectable.

**Table 1:** Patient characteristics and laboratory data

	group A	group B	*P *value
	n = 31	n = 67	
**patient characteristics**			
Age (years)	79.09 ± 3.49	61.69 ± 6.37	< .0001
Gender			0.14
male	16	45	
female	15	22	
Body mass index	23.04 ± 3.85	22.82 ± 3.84	0.8
Comorbidity			
Hypertension	13	20	0.24
Hyperlipoproteinemia	5	7	0.42
Diabetes mellitus	5	12	0.83
Cardiac disease	2	5	0.86
Plumonary disease	1	1	0.57
Chronic renal disease	1	0	0.14
cerebral disease	0	3	0.23
Other	1	0	0.14
Preoperative biliary drainage			
Yes	24	48	0.55
No	7	19	
Diagnosis			
Pancreatic adenocarcinoma	8	31	0.08
Cholangiocarcinoma	15	22	
Ampullary carcinoma	5	3	
Intraductal papillary mucinous neoplasm	2	4	
Duodenal carcinoma	0	3	
Gastric carcinoma	1	0	
Duodenal invasion of colon carcinoma	0	1	
Other	0	3	
			
**Laboratory data**			
Hemoglobin	12.20 ± 1.81	14.24 ± 15.42	0.46
Total bilirubin	6.90 ± 7.10	4.85 ± 5.01	0.16
Amylase	91.48 ± 44.93	153.48 ± 234.91	0.15
Hemoglobin A1c	5.70 ± 2.08	6.29 ± 2.03	0.32

### Operative outcomes

The 31 PDs (standard PD in 6 patients (19.4%), SSPPD in 7 (22.6%) and PPPD in 18 (58.1%)) performed in group A included Cholangiocarcinoma in 15 (48.4%) patients, Pancreatic adenocarcinoma in 8 (25.8%), Ampullary carcinoma in 5 (16.1%), Intraductal papillary mucinous neoplasm in 2 (6.5%), and Gastric carcinoma in 1 (3.2%). The 67 PDs (standard PD in 20 patients (29.9%), SSPPD in 10 (14.9%) and PPPD in 37 (55.2%)) performed in group B included Pancreatic adenocarcinoma in 31 (46.3%) patients, Cholangiocarcinoma in 22 (32.8%), Intraductal papillary mucinous neoplasm in 4 (6.0%), Ampullary carcinoma in 3 (4.5%), Duodenal carcinoma in 3 (4.5%), Duodenal invasion of colon carcinoma in 1 (1.5%), and Other in 3 (4.5%).

The mean surgical time in group A was 408.1 ± 73.47 min, which was significantly shorter than that in group B (P = .04). Blood loss and blood transfusion were not significantly different between groups (Table 2).

**Table 2:** Operative outcomes

	group A	group B	*P *value
	n = 31	n = 67	
Type of pancreaticoduodenectomy			
PPPD	18	37	0.44
SSPPD	7	10	
PD	6	20	
Pancreatic texture			
soft	26	47	0.04
hard	5	27	
Tumor size (mm)	27.17 ± 12.18	30.59 ± 13.64	0.25
Pancreatic duct diameter (mm)	3.61 ± 3.03	4.03 ± 2.94	0.58
Pancreatic drainage			
internal	12	28	0.77
external	19	39	
Surgical time (min)	408.1 ± 73.47	461.76 ± 84.98	0.003
Blood loss (ml)	948.58 ± 468	1225.5 ± 925.82	0.12
Blood transfusion (ml)	209.33 ± 425.62	231.64 ± 510.57	0.84
Portal vein resection			
Yes	1	8	0.16
No	30	59	

PD = pancreaticoduodenectomy; SSPPD = substomach preserving pancreaticoduodenectomy; PPPD = pylorus preserving pancreaticoduodenectomy.

### Pathologic results

The American Joint Committee on Cancer (AJCC) cancer staging distribution was 4 (13.3%) stage 0, 9 (30%) stage I, 16 (54.3%) stage II, and 1 (3.3%) stage III in group A and 2 (3.2%) stage 0, 25 (39.1%) stage I, 34 (53.7%) stage II, and 3 (6.7%) stage III in group B. The mean tumor size was 27.17 ± 12.18 mm in group A and 30.59 ± 13.64 mm in group B (P = .25). The distribution of histological grades in group A was as follows: 12 (38.7%) well differentiated, 9 (29%) moderately differentiated, 4 (12.9%) poorly differentiated, 3 (9.7%) papillary, and 3 (9.7%) other. In group B, it was as follows: 14 (21%) well differentiated, 27 (40.6%) moderately differentiated, 13 (20%) poorly differentiated, 7 (10.7%) papillary, and 6 (9.1%) other (Table 3).

**Table 3:** Pathological results

	group A	group B	*P *value
	n = 31	n = 67	
AJCC T stage			0.28
is	4	2	
1	3	11	
2	10	16	
3	12	32	
4	1	3	
AJCC Nodal status			0.87
N0	16	33	
N1	14	31	
AJCC Stage			0.34
0	4	2	
IA	1	9	
IB	8	16	
IIA	3	6	
IIB	12	27	
III	1	2	
Grade of neoplasm			0.43
well	12	14	
moderately	9	27	
poorly	4	13	
papillary	3	7	
other	3	6	
			

AJCC = The American Joint Committee on Cancer

### Postoperative outcome

The mean postoperative stay in group A was 25 ± 16.46 days, which was longer than that in group B (21.54 ± 10.32 days), but the difference was not significant. One in-hospital death occurred in group A on postoperative day 22. There was no significant difference in mortality rate between group A and group B (3.2% vs. 0%, P = .14). The overall complication rate was 50.0% (54.8% in group A and 46.3% in group B; P = .43). The most common complication was pancreatic fistula (32.7%). The incidence of pancreatic fistula was similar in group A and group B (38.7% vs. 29.9%, respectively; P = .38). There was also no significant difference in the occurrence of other complications: delayed gastric emptying, liver abscess, wound infection, intraabdominal bleeding, respiratory insufficiency, intraabdominal collection, sepsis, bile leakage, or gastrointestinal bleeding. There are summarized in Table 4.

**Table 4:** Postoperative outcomes

	group A	group B	*P *value
	n = 31	n = 67	
**Complications**			
Pancreatic fistula	12	20	0.38
Grade A	5	5	
Grade B	6	15	
Grade C	1	0	
Delayed gastric emptying	2	4	0.93
Liver abscess	0	1	0.49
Bowel obstruction	0	0	1
Wound infection	5	5	0.19
Intraabdominal bleeding	1	2	0.95
Respitory insufficiency	4	3	0.13
Intraabdominal collection	3	3	0.32
Sepsis	1	0	0.14
Bile leakage	1	0	0.14
Gastrointestinal bleeding	0	1	0.49
**Postoperative outcomes**			
Postoperative hospital stay (day)	25 ± 16.46	21.54 ± 10.32	0.22
Mortality	1	0	0.14
Morbidity	17	31	0.43

### Long-term survival

The mean follow up was 15.8 months in group A (median: 12.0 months; range, 0.8-65.4 months) and 23.3 months in group B (median: 18.8 months; range, 1.3-59.5 months). The 1- and 3-year survival rates were 70.0% and 50.5%, respectively, in group A and 84.8% and 65.9%, respectively, in group B. There was no statistically significant difference between the two groups using the log-rank test (P = .10).

## Discussion

There have been many cases of PD since the first report by Wipple et al. in 1935 [13]. However, the incidence of postoperative complications is still 30%-60%[14-16]. PD may cause considerable complications, including pancreatic fistula, intra-abdominal bleeding, intra-abdominal abscess, sepsis and organ failure, and requires high-quality techniques and management methods in the perioperative period. Though PD is performed in many hospitals, its associated morbidity and mortality rates are high. However, the rate of morbidity and mortality associated with PD has decreased, especially in high-volume centers [6-8]. Complications tend to be lower in institutions that perform more than a certain number of surgeries and have more than a certain number of staff medical specialists; such institutions provide better management of complications, which provides the strength of the recommendation. It was suggested that PD was contraindicated in most elderly patients, because such aggressive surgery would result in perioperative complications. Yeo et al [17] noted that age appears to be an important predictor of death in low-volume centers but not in high-volume centers. Several studies reported that age was not an independent risk factor for perioperative mortality and morbidity following PD [11,18]. Since PD is the only chance these patients have for a cure, we suggest that PD is justified, even in the elderly. If appropriate management of elderly patients is provided, the safety of perioperative management will be secured in high-volume centers. Therefore, it has been reported that patients should not be excluded from PD due to age [10].

In our institution, we also investigated the safety of elderly patients following PD. Patients aged 75 years and older (group A) had remarkably similar outcomes compared to younger patients (group B), with no differences in patient characteristics and preoperative laboratory data, excluding albumin. Moreover, there was no difference in morbidity and mortality between groups. The mean surgical time for group A was significantly shorter than that for group B (P < 0.01). It was suggested that this was because a portal vein resection was done for only 1 of 31 patients (3.2%) in group A, which was less than that done in group B (11.9%), although this difference was not significant. Pancreatic fistula is the most threatening complication of PD. In the literature, the rates of pancreatic fistula range from 5% to 40%[2-5]. In the present study, the incidence of pancreatic fistula was similar between older and younger patients (45.1% vs. 29.9%, respectively; P = .14), though there was a tendency for increased normal pancreatic texture (soft pancreas) in older patients (P = .04). Several reports compared external drainage and no-stent procedures and found that the incidence of fistula was significantly lower for external drainage [3,19]. The normal pancreas preserves exocrine function, and its main pancreatic duct is narrow. Thus, one cannot rule out the possibility of injury during surgical manipulation. The diameter of the pancreatic duct is approximately 1-2 mm in the normal pancreas, and postoperative swelling can develop temporarily that can result in stenosis. Thus, stent placement is considered essential at our institution. It is thought that placement of an external drain can minimize the leakage from a branch of remnant pancreatic duct. The external drain reduces the stress at the anastomotic site by a pressure gradient and minimizes the outflow into the branch of the pancreatic duct. We placed an external drain for the normal pancreas. We suggest that an external drain reduces the incidence of pancreatic fistula in a normal pancreas.

Older patients also had similar lengths of postoperative hospital stay compared with younger patients. The rate of overall survival in older patients tended to be lower compared with younger patients, though the difference was not statistically significant. Recently, most patients with pancreatic carcinoma receive adjuvant chemotherapy [20]. It is difficult to compare older and younger patients, as we did not manage some older patients after surgery, because we considered the side effects of chemotherapy due to their age, especially for those over 80 years of age. Aloia et al report delayed recovery after PD [21]. Because patient age was independently associated with a decreased likelihood of receiving adjuvant therapy by multivariate analysis, we suggest that it might be better to avoid adjuvant chemotherapy after surgery for elderly patients. Except for this matter, our data showed no significant differences in postoperative morbidity and mortality between older and younger PD patients.

The results of several series [22-26] suggest that age is unrelated to morbidity and mortality and that PD can be performed safely if it is provided by appropriate indication and management. Our study also addresses the safety of PD management in elderly patients and supports these opinions.

## Conclusion

In conclusion, it cannot be ignored that the elderly population is getting larger. We must investigate the management of elderly patients after PD and prepare further for more experiences of PD. If appropriate surgical management is provided to elderly patients, we suggest that PD will lead to no adverse effects after surgery, and PD can be performed safely in elderly patients. We conclude that age should not be a contraindication to PD.

## Competing interests

The authors declare that they have no competing interests.

## Authors' contributions

YI designed methods and carried out the instructions, analyzed the data, interpreted the results and drafted the manuscript. All authors read and approved the final manuscript.
